# High Fat Diet Alters Gastrointestinal Function and Results in Reduced Enteric Neuron Density

**DOI:** 10.1111/nmo.70470

**Published:** 2026-10-18

**Authors:** Marta Chavero‐Pieres, Maria Francesca Viola, Zheng Wang, Marcello Delfini, Iris Appeltans, Naomi Fabre, Guy Boeckxstaens, Nathalie Stakenborg

**Affiliations:** ^1^ Laboratory for Intestinal Neuro‐Immune Interaction, Translational Research Center for Gastrointestinal Disorders, Department of Chronic Diseases Metabolism and Ageing, KU Leuven Leuven Belgium; ^2^ Developmental Biology of the Immune System, Life & Medical Sciences (LIMES) Institute University of Bonn Bonn Germany

**Keywords:** constipation, enteric nervous system, gastrointestinal tract, high‐fat diet, obesity

## Abstract

**Introduction:**

Obese patients suffer from cardiovascular, metabolic and gastrointestinal disorders, including constipation, proposed to result from high fat diet (HFD)‐induced enteric neuron loss. However, these studies used high‐fat purified diets, containing reduced amounts of fiber compared to non‐purified diets, known to alter gut physiology and microbiome composition. Therefore, we evaluated the effect of purified HFD‐induced obesity on enteric neuron density and gastrointestinal transit compared to its proper control diet, that is, a purified control diet.

**Methods:**

Wild type mice were fed a standard chow diet, purified low fat diet (LFD) or HFD. Colonic transit was measured via glass bead expulsion following 12, 18, and 24 weeks of diet. Colonic muscularis externa was collected for immunofluorescent stainings of enteric neurons.

**Results:**

Starting from 18 weeks of HFD, a decline in enteric neuron number was observed compared to LFD. In addition, delayed colonic transit was observed in HFD‐fed mice when compared to LFD‐fed animals after 18 weeks of exposure.

**Conclusions:**

Our study demonstrates that prolonged exposure to a HFD results in a reduced enteric neuron density and delayed colonic transit, and underscores the need to use a proper control diet when evaluating the impact of a HFD on gut physiology.

## Key Points

- An appropriate reference diet is of key importance to investigate diet‐induced obesity. A prolonged exposure to high‐fat diet is needed to induce obesity‐associated alterations in the enteric network and colonic transit.

## Graphical abstract

Our study shows that a proper control diet is essential to study the effect of high fat diet (HFD) on gut motility and enteric neuron density. Prolonged exposure to HFD (> 18 weeks) causes an increased colonic transit as well as enteric neuron loss compared to low fat diet‐fed mice.

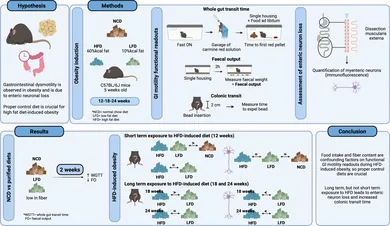

## 1 Introduction

Current sedentary lifestyles are leading to an enormous rise in the prevalence of obesity over the last decades, reaching pandemic levels. Consequently, this has led to a substantial increase in the incidence of obesity‐associated metabolic disorders including cardiovascular disease, Type 2 diabetes mellitus, fatty liver disease, and some types of cancer, placing a growing health‐care burden [1]. Accumulating evidence suggests that high‐fat diets (HFD) and obesity are linked to alterations in gastrointestinal motility. Amongst other gastrointestinal complaints, constipation is often reported in obese individuals [2, 3]. It is however still unclear if such gastrointestinal problems result from obesity per se, the use of multiple medications, dietary or lifestyle factors or can be attributed to neuronal loss in the enteric nervous system (ENS).

Obesity is a state of chronic low‐grade inflammation associated with increased levels of oxidative stress and intestinal barrier dysfunction, which induces changes in the cell microenvironment and tissue damage [4]. Previous studies have demonstrated the loss of enteric neurons in the colonic myenteric plexus in animal models of diet‐induced obesity [5, 6, 7], involving multiple factors. Exposure to elevated levels of free fatty acids has been reported to trigger apoptosis of enteric neuronal cells by inducing mitochondrial dysfunction and endoplasmic reticulum stress [6, 8, 9]. In addition, a synergistic effect of increased levels of intraluminal LPS and palmitate can induce a reduction of neuronal abundance via TLR4 signaling in myenteric neurons [5]. Furthermore, increased levels of neuronal pyroptosis have been detected in obese individuals [10], affecting mainly nitrergic neurons which are responsible for regulating the descending inhibitory reflex during peristalsis [11].

Of key importance, diets with high fat content used for the induction of obesity in animal models are generally made with refined, purified ingredients and have a defined composition. Nonetheless, numerous studies compare purified HFDs with unpurified normal chow diets (NCD) as their control. However, the fiber content in purified HFDs is significantly lower than that of NCD diet. Since fibers are known to significantly alter gut physiology and microbiome composition, it is key to use an ingredient matched, purified control diet in order to avoid confounding effects attributable to differences in diet composition. In this study, we therefore investigated if HFD‐induced obesity results in a reduced number of enteric neurons. To this end, mice were exposed to a purified (low fat or high fat) versus an unpurified diet for up to 24 weeks. Whole gut transit and colonic bead expulsion were performed to assess GI motility. Enteric neuronal density was assessed via quantification of enteric neurons.

## 2 Materials and Methods

### 2.1 Mice

All animal experiments were performed following the guidelines set by the European Community Council and were approved by the Animal Care and Animal Experiments Committee of the Medical Faculty of the KU Leuven (P043/2020). Male C57Bl/6j (3 weeks old) were purchased from Janvier (Mayenne, France). Mice were kept under a 12 h/12 h dark/light cycle, at a temperature of 20°C–22°C with humidity ranging from 45% to 70% and provided with food and water ad libitum.

### 2.2 High Fat Diet‐Induced Obesity

Obesity was induced by a purified high fat diet with 60% calories of fat (HFD, D12492, Research Diets Inc., New Brunswick, USA) starting at the age of 5 weeks in male WT C57Bl/6j mice during 12–24 weeks. Age matched control animals were administered either a normal chow diet (NCD) or a purified low fat diet (LFD, D12450J, Research Diets Inc., New Brunswick, USA). LFD and HFD were matched for protein (casein and L‐Cystine), sucrose, soybean oil and fiber (cellulose), but differed in their corn starch and lard content (Figure 1a). Body weight of LFD‐ and HFD‐fed animals was checked weekly (Figure S1A). Blood glucose measured from LFD‐ and HFD‐fed animals is shown at baseline and at 12, 18, and 24 weeks following the respective diets in Figure S1B.

**FIGURE 1:**
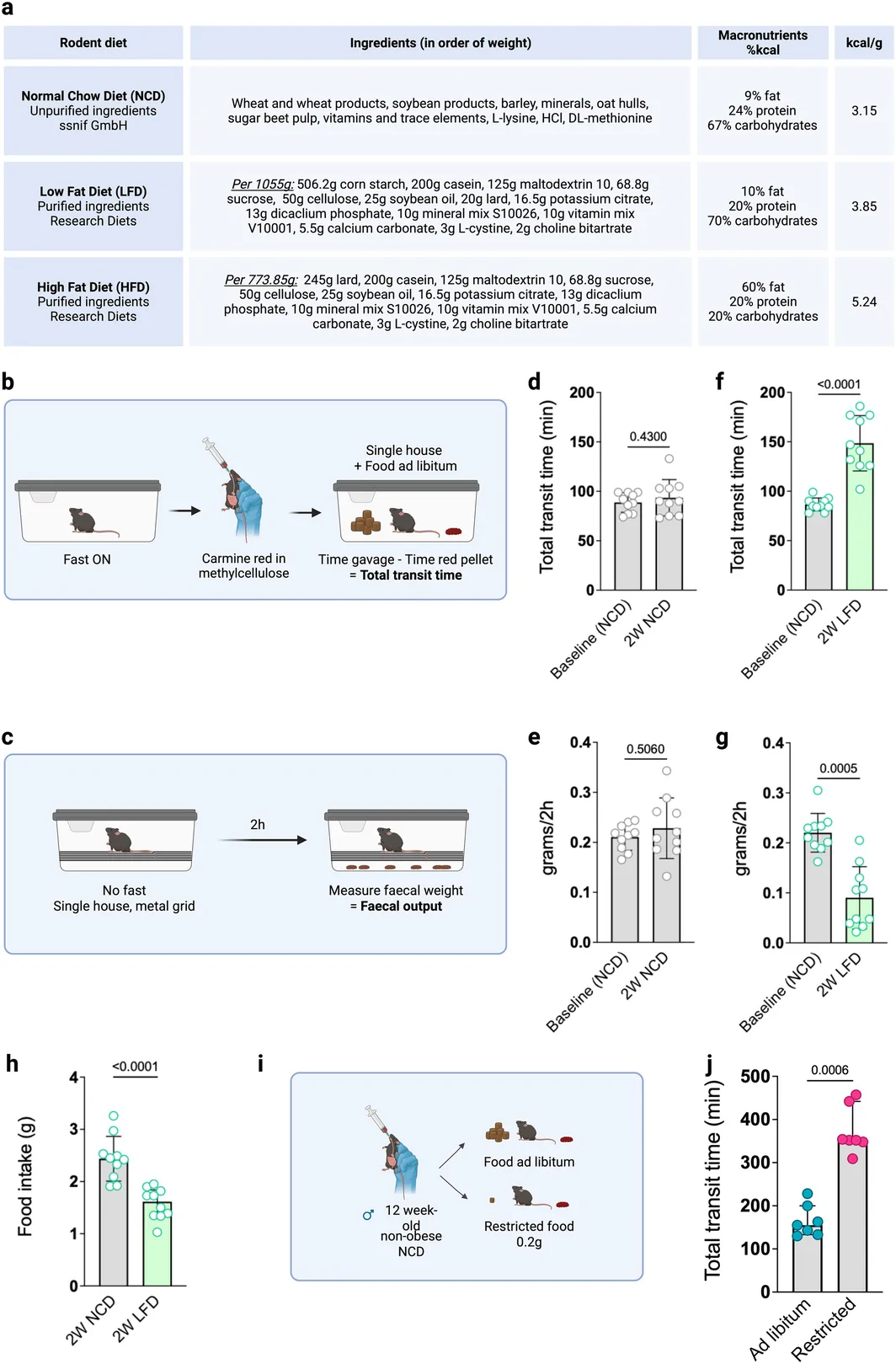
Diet composition and food intake significantly affect gastrointestinal function. (a) Overview of ingredient and macronutrient composition of diets used in the present study, including normal chow diet (NCD), low fat diet (LFD) and high fat diet (HFD). (b) Schematic representation of the experimental protocol to determine whole gut total transit time (WGTT) by carmine red gavage. (c) Schematic representation of the experimental protocol to determine fecal output. (d) WGTT of mice at baseline (NCD) and at 2 weeks after NCD feeding (*n* = 10). (e) Fecal output of mice at baseline (NCD) and at 2 weeks after NCD feeding (*n* = 10). (f) WGTT of mice at baseline (NCD) and at 2 weeks after LFD feeding (*n* = 10). (g) Fecal output of mice at baseline (NCD) and at 2 weeks after LFD feeding (*n* = 10). (h) Food intake of mice following 2 weeks of NCD or LFD feeding (*n* = 10/group). (i) Schematic representation of the experimental protocol to determine the effect of food intake on the assessment of WGTT. (j) WGTT of mice with food ad libitum or restricted food (0.2 g) in 12‐week adult non‐obese mice fed a normal chow diet (*n* = 7/group). (d–g) Data are expressed as mean ± SD and were analyzed by paired *t*‐test. (h) Data are expressed as mean ± SD and were analyzed by unpaired *t*‐test. (i) Data are expressed as median ± IQR and were analyzed by Mann–Whitney test. Illustrations were created using Biorender.com.

### 2.3 Whole Gut Transit Time (WGTT)

WGTT was evaluated using carmine red gavage as previously described [12]. In brief, mice were acclimatized 2 days prior to the assessment by a saline gavage and a period of 3 h in single‐housing. After an overnight fast, mice received a gavage of a 6% w/v solution of carmine red (Sigma) in sterile methyl cellulose (0.5%) and were then placed in single‐housing. Mice were then monitored every 15 min until the appearance of the first red pellet. The time from the carmine red gavage until the appearance of the first red pellet was recorded as WGTT.

To evaluate the effect of food intake during the WGTT assessment, we determined WGTT in mice fed NCD ad libitum or restricted to 0.2 g. In brief, all mice were fasted overnight, then gavaged with carmine red, and then either given a 0.2 g food ball or free access to food as described previously [13]. The food restriction lasted for the whole assay; after the experiment, all animals received food and water ad libitum.

### 2.4 Fecal Output

For the assessment of fecal output, mice were placed on a metal grid during 2 h, with continuous access to food and water. The fecal pellets produced within this 2‐h period were collected and weighed, and the total weight was deemed as the fecal output for the given 2‐h interval.

### 2.5 Colonic Bead Expulsion Test for Determination of Distal Colonic Transit

Distal colonic transit was measured by placing a 3 mm glass bead 2 cm proximally to the anal opening as previously described [7]. Briefly, mice were anesthetized with isoflurane in order to facilitate the precise placement of the spherical glass bead. Subsequently, mice were housed individually in plastic cages and monitored for bead expulsion. Distal colonic transit was determined by measuring the time elapsed between bead placement and expulsion.

### 2.6 Histology and Immunofluorescence

The colon was carefully dissected, flushed using Hank's Balanced Salt Solution (HBSS) (Gibco, Waltham, USA) supplemented with fetal calf serum (FCS, 3%, Lonza, Basel, Switzerland) and HEPES (1.8%; (Gibco, Waltham, USA)) and then pinned on a sylgard plate. After removing mesenteric fat, the tissue was longitudinally opened and pinned with the muscle layer facing upwards. The muscle layer was carefully peeled from the submucosa, pinned flat on the sylgard plate and then fixed for 30 min in 4% paraformaldehyde (PFA 4%; Sigma‐Aldrich, Saint Louis, USA). Subsequently, the tissue was permeabilized in PBS supplemented with Triton X‐100 (0.3%; permeabilization buffer; ThermoFisher Scientific, Waltham, USA) for 2 h at room temperature, followed by blocking with permeabilization buffer containing Donkey serum (5%; Jackson Immunoresearch, Westgrove, USA) and Bovine serum albumin (BSA, 5%; blocking buffer; Sigma‐Aldrich, Saint Louis, USA) for 2 h at room temperature.

For immunostaining, the tissue was incubated with primary antibodies diluted in blocking buffer overnight at 4°C. After washing three times with PBS, the tissue was incubated with secondary antibodies diluted in blocking buffer for 2 h at room temperature. Following additional washing steps with PBS, the samples were mounted using SlowFade Diamond Antifade mounting medium (Thermofisher Scientific, Waltham, USA) and imaged using a Zeiss LSM880 confocal microscope (Oberkochen, Germany). Ten regions were selected per animal at random. Quantification of the HuC/D‐positive neurons was done in a blinded fashion for each image. HuC/D‐positive enteric neurons were identified based on their characteristic immunoreactivity and morphology, so each morphologically identifiable positive soma was counted as a single neuron. All antibodies used are listed in Table S1. Negative controls (incubated with secondary but no primary antibody) were included for analysis (data not shown).

### 2.7 Statistics

Statistical analysis was performed using Prism 10 (GraphPad Software, Boston, USA). The data are represented as mean ± SD or median ± interquartile range (IQR) depending on normality. Data were analyzed using (un)paired *t*‐tests or one‐way ANOVA (followed by Tukey's multiple comparisons) when the data were normally distributed. Otherwise, the data were analyzed using Mann–Whitney *U* test or Kruskal–Wallis test (followed by Dunn's multiple comparisons). Grubbs outlier test was used to identify and exclude statistical outliers. A significance threshold of *p* value < 0.05 was applied to determine statistical significance.

## 3 Results

### 3.1 Differences in Diet Composition and Food Intake Affect Whole Gut Transit Time

To determine the effect of the diet composition on gastrointestinal motility, we assessed WGTT (Figure 1b) and fecal output (FO) (Figure 1c) in male C57Bl/6j mice at baseline (before randomizing to different diets) and after 2 weeks administration of normal chow diet (NCD) or a purified low fat diet (LFD). While animals receiving NCD maintained similar values of WGTT and fecal output after 2 weeks (Figure 1d,e), mice subjected to a purified LFD, poor in fibers, presented a significant delay in WGTT and a reduced fecal output indicative of constipation (Figure 1f,g), suggesting that reduced fiber content in purified diets rapidly alters gastrointestinal function. These findings show the importance of using matched diets during transit studies and highlight the key role of fiber content in gastrointestinal function.

In addition to the content of dietary fibers, allowing food ingestion during the assessment of WGTT may be a source of variability further hindering comparison between studies. Therefore, we quantified food intake during the WGTT measurement in mice that received either NCD or a purified LFD for a period of 2 weeks. Notably, a reduced amount of food intake was observed in the LFD compared to the NCD group (Figure 1h). Next, to evaluate the effect of food intake during the WGTT assessment, we determined WGTT in mice fed NCD ad libitum or restricted to 0.2 g (Figure 1i). WGTT was significantly delayed in animals that received a restricted volume of food compared to feeding ad libitum (Figure 1j), suggesting that food intake and the type of diet significantly affect WGTT.

### 3.2 12 Weeks HFD Does Not Induce Altered Enteric Neuronal Density or Colonic Transit

Next, we evaluated the effect of diet‐induced obesity on gastrointestinal motility and enteric neuronal abundance. To this end, we subjected animals to (1) a standard NCD (2) a purified LFD and (3) a purified HFD (Figure 1a). As we showed that WGTT is significantly affected by diet and food intake, we evaluated gastrointestinal function by performing two other functional tests, namely fecal output and colonic bead expulsion test.

As shown in Figure 2a, fecal output was significantly reduced in mice fed for 12 weeks with LFD and HFD compared to NCD animals (Figure 2a). Also, higher bead expulsion times were observed in both LFD and HFD fed mice, reaching statistical significance in the HFD but not in the LFD fed group compared to animals receiving NCD (Figure 2b,c). Yet, no changes in fecal output or in the bead expulsion test were observed between animals subjected to LFD and HFD, emphasizing the importance of a purified diet as proper control diet.

**FIGURE 2:**
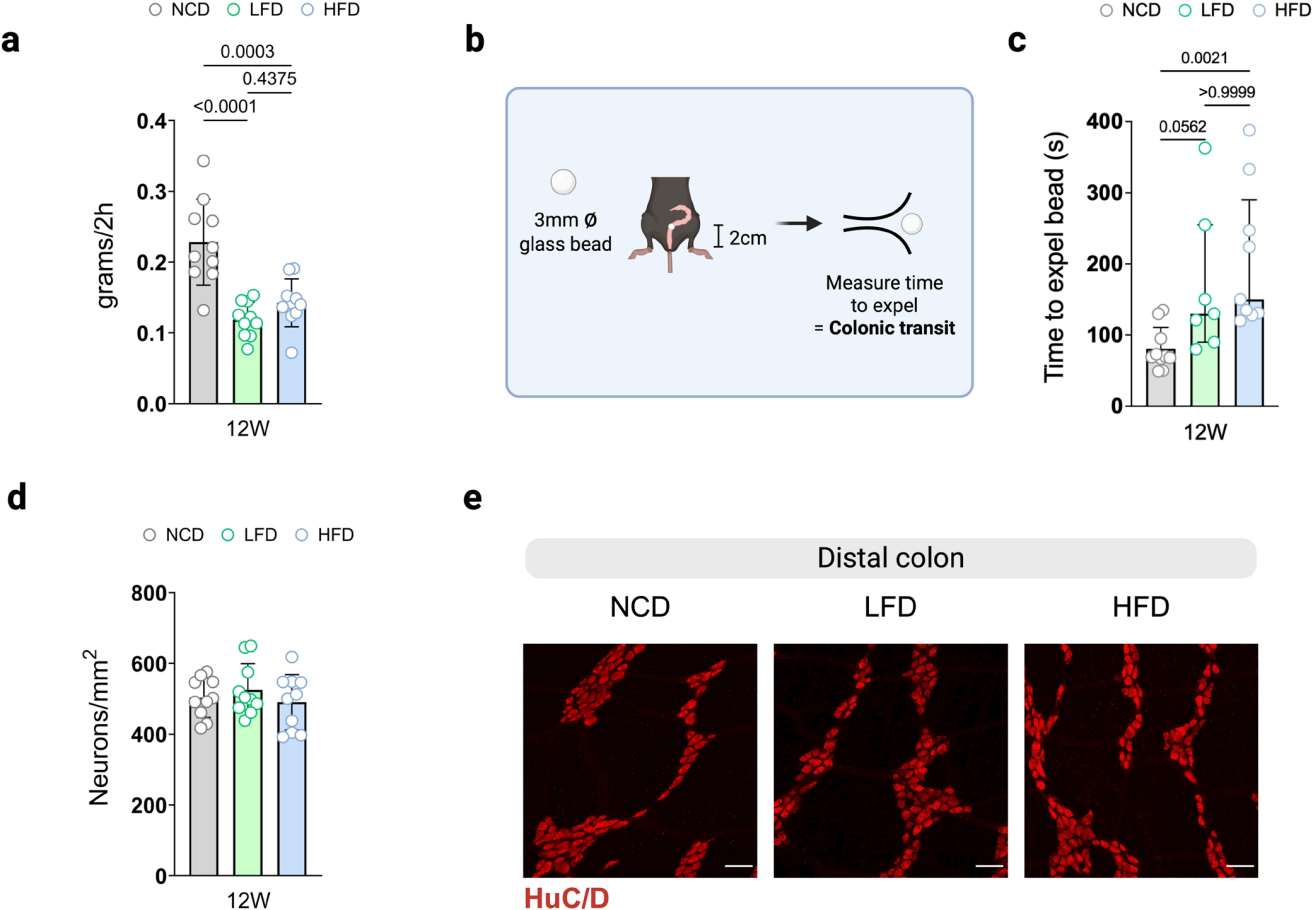
No loss of neuronal abundance or alterations in gastrointestinal function are detected after 12 weeks of high fat diet (HFD) feeding. (a) Fecal output after 12 weeks of administration of normal chow diet (NCD), purified low fat diet (LFD) or HFD (*n* = 10/group). (b) Schematic representation of the experimental protocol to determine colonic transit by the bead expulsion test. (c) Colonic transit after 12 weeks of administration of NCD, purified LFD or HFD (*n* = 7–10/group). (d) Neuronal density, as determined by the number of HuC/D+ soma per mm^2^ of tissue, in the myenteric plexus of the distal colon of mice fed a NCD, purified LFD and HFD for 12 weeks (*n* = 10/group). (e) Representative immunohistochemical stainings of distal colon whole mount muscular preparations showing enteric neurons (red, HuC/D+) of mice receiving NCD, purified LFD and HFD for 12 weeks. Scale bar is 50 μm. Data are expressed as mean ± SD (a, d) or median ± IQR (c) and were analyzed by ANOVA followed by Tukey's multiple comparisons test (a, d) or Kruskal‐Wallis test using Dunn's multiple comparisons test (c). Illustrations were created using Biorender.com.

Next, we quantified neuronal density in the myenteric plexus of the distal colon after 12 weeks feeding of the respective diets. In line with the functional tests, no changes were observed in the number of neurons in the distal colon between animals receiving a LFD and HFD (Figure 2d,e). Moreover, no differences in enteric neuronal density were observed between animals receiving purified LFD or HFD and those receiving unpurified NCD, despite differences in colonic transit, suggesting that the observed transit alterations cannot be solely explained by changes in enteric neuronal density.

### 3.3 Long Term Administration of HFD Induces Reduced Enteric Neuronal Abundance and First Signs of Altered Motility

To assess if more prolonged exposure to a HFD is required to induce a reduced enteric neuron density, mice were exposed to LFD or HFD during 18 and 24 weeks. Of interest, after 18 weeks, neuronal density was significantly reduced in the distal colon of HFD fed mice, further decreasing after 24 weeks (Figure 3a–d). Fecal output remained unchanged at 18 and 24 weeks between purified LFD and HFD fed mice (Figure 3e,f). On the other hand, there was delayed colonic transit after 18 weeks of HFD exposure when compared to LFD and NCD mice. However, no significant differences were observed between the LFD and HFD fed groups after 24 weeks of dietary intervention (Figure 3h). On an individual‐animal level we observed that 5 out of 10 animals had expulsion times above the threshold set by the 95th percentile of expulsion time values in animals receiving a LFD for 18 weeks (Figure 3g), while at 24 weeks of dietary intervention 3 out of 10 HFD fed animals displayed delayed colonic transit times over 400 s, of which 2/3 mice also presented with increased bead expulsion times at 18 weeks, suggesting a slower colonic motility due to HFD exposure in a subgroup of mice.

**FIGURE 3:**
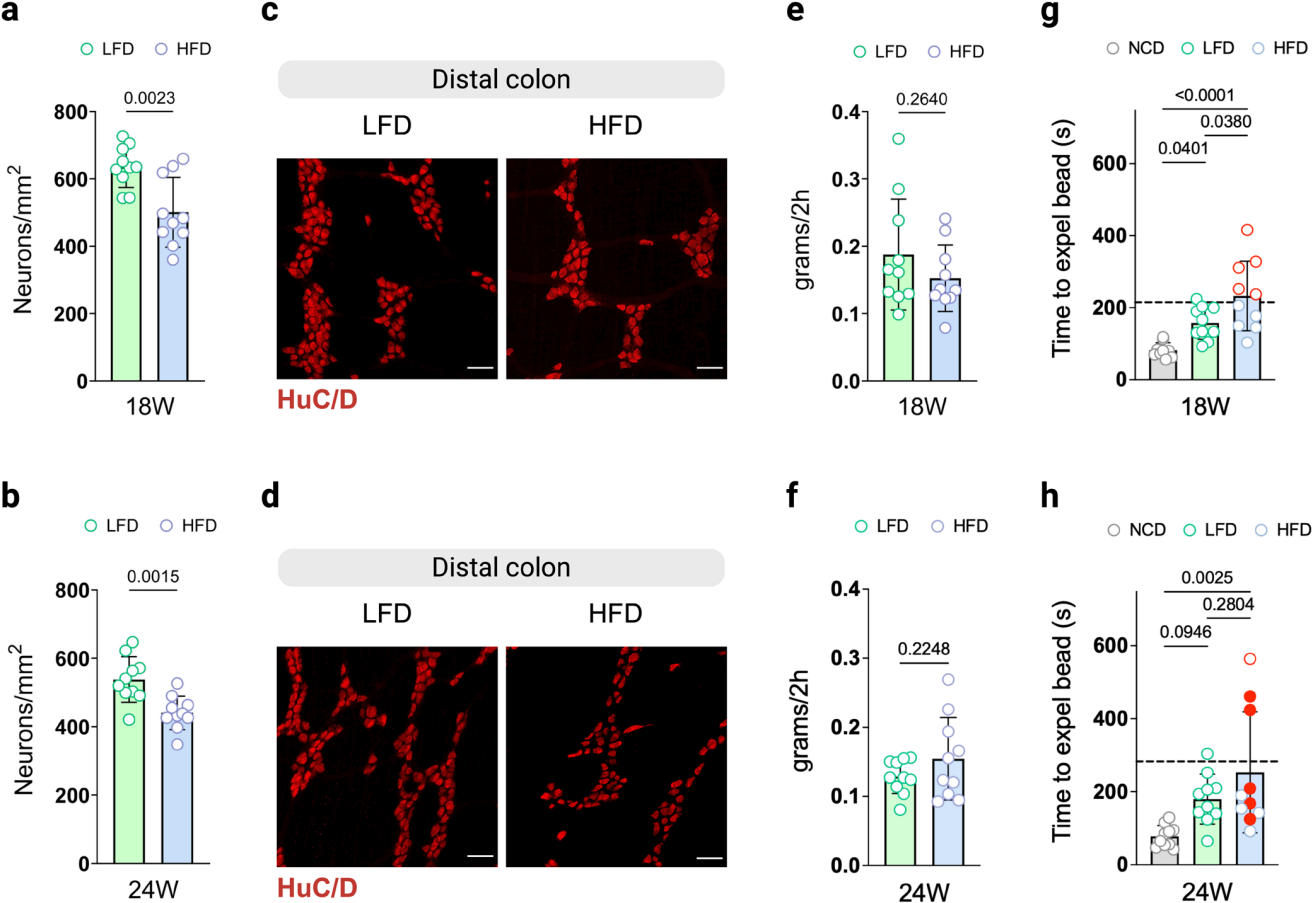
Long‐term high fat diet (HFD) feeding is accompanied by reduced enteric neuron density and alterations in colonic motility. (a‐b) Neuronal density in the myenteric plexus of the distal colon of mice fed a purified low fat (LFD) and purified HFD for 18 (a) and 24 (b) weeks (*n* = 10/group). (c‐d) Representative immunohistochemical stainings of distal colon whole mount muscular preparations showing enteric neurons (red, HuC/D+) of mice receiving normal purified LFD and HFD for 18 (c) and 24 (d) weeks. Scale bar is 50 μm. (e‐f) Fecal output after 18 (e) and 24 (f) weeks of administration of purified LFD or HFD (*n* = 10/group). (g‐h) Colonic transit after 18 (g) and 24 (h) weeks of administration of normal chow diet (NCD), purified LFD or HFD (*n* = 9–10/group). Individual points with red borders depict animals with delayed gastrointestinal function according to the threshold set by the 95th percentile of values obtained in animals fed a LFD (g‐h). Individual points highlighted in red depict animals that presented delayed gastrointestinal function at the 18 weeks timepoint (h). Data are expressed as mean ± SD (a, b, e–h) and were analyzed by unpaired *t*‐test (a, b, e, f) and one way ANOVA with Tukey's multiple comparisons test (g, h).

## 4 Discussion

Here, we investigated the impact of diet‐induced obesity on enteric neuron density and gastrointestinal function. We showed that a low‐fiber purified diet has a strong impact on gastrointestinal transit, emphasizing the importance of using a matched purified diet as control. Moreover, only prolonged high‐fat diet exposure was associated with reduced myenteric neuronal density and transient alterations in colonic transit, stressing the need for further research to elucidate the mechanism underlying changes in neuronal density and colonic motility in obesity. The choice of control diet in animal studies holds significant importance when investigating the effect of diet‐induced obesity and its related disorders. In particular, careful selection of a relevant control diet becomes crucial when examining metabolic parameters and intestinal physiology in animal models. Nevertheless, studies often compare purified HFDs to standard unpurified NCDs, which are distinct in composition and may lead to different phenotypes [14, 15], thereby confounding data interpretation. There are many compositional differences between purified diets and unpurified chow diets. Yet, their fiber content is likely the most substantial difference between diets, both in quantity and composition. While chow diets contain up to 20% of insoluble and 5% of soluble fibers, in purified diets its content is limited to a total of 5% which is derived solely from an insoluble source [14, 16]. Unlike its soluble counterpart, insoluble fibers are non‐fermentable and therefore intestinal bacteria are unable to metabolize them into short‐chain fatty acids (SCFA), which play a crucial role in maintaining intestinal homeostasis. As seen in mice exposed to purified diets, either low‐fat or high‐fat, such reduction or absence of fibers results in decreased levels of SCFAs, reduced bacterial diversity, increased intestinal permeability and a reduction in size of the small intestine and colon, irrespective of the amount of dietary fat [14, 15]. Altogether, this suggests that not only the high‐fat content but also the lack of fibers may drive some of the alterations observed in mice subjected to HFD. Indeed, we demonstrated that WGTT and fecal output of mice on a purified diet are significantly different from that of mice on a normal chow diet even after only 2 weeks of exposure. Moreover, our findings demonstrate that food intake has a strong impact on WGTT, which shows the complexity of studying the functional implications of diet and food intake on gastrointestinal transit. Hence, it is key to administer a purified low‐fat diet as a control diet in order to specifically investigate the role of dietary fat in disease conditions, particularly in studies concerning the intestinal tract where fibers play a crucial role in maintaining gastrointestinal health.

When compared to animals under NCD, mice exposed to a HFD for 12 weeks had a reduced fecal output and increased bead expulsion times. However, compared to animals subjected to a purified LFD, no differences in fecal output nor in colonic transit were observed, suggesting that when using a matched purified control diet, HFD exposure does not alter colonic motility. As previously discussed, colonic alterations may result from the distinct composition of purified diets, regardless of their fat content. Consistent with these findings, previous studies comparing a purified high‐fat diet with a purified low‐fat diet showed no differences in colonic transit time after 12 weeks [5]. However, when unpurified high‐fiber normal chow diets are used as control, a delayed colonic transit time and reduced fecal output were reported in HFD‐fed animals [6, 7, 17]. Of interest, supplementation with soluble fiber oligofructose rescued the HFD‐induced delay in transit [7]. Taken together, these results further support our findings and indicate that the previously reported delayed intestinal transit in HFD mice, compared to mice on NCD, is at least in part due to the reduced fiber content. Yet, the literature contains conflicting findings regarding altered colonic transit after 12 weeks in diet‐induced obesity, which may be due to differences in the distance of insertion to the anal opening, the diameter of the inserted glass bead or the obesogenic diet used. Interestingly, studies performed by Reichardt and colleagues report contrasting results when using a HFD or a Western diet. While also widely used to induce obesity and obesity‐related comorbidities, a Western diet generally contains a lower fat content than routinely used in HFDs but higher levels of sucrose and salt [7, 18]. Strikingly, animals subjected to a HFD presented accelerated colonic transit after 12 weeks of exposure when compared to a purified low‐fat control, which was attributed to upregulation and enhanced signaling of acetylcholine and serotonin [19]. Conversely, mice subjected to a Western diet displayed delayed colonic transit associated with reduced numbers of myenteric neurons [5]. Collectively, these data underscore the urgent need for a deeper understanding of how different diets may influence the observed phenotype in animal models of diet‐induced obesity and further highlights the importance of standardizing the experimental diets used prior to drawing conclusions.

Obesity is a long‐term, chronic health condition often leading to a gradual development of its associated comorbidities over time, such as cardiovascular disease, dyslipidaemia, Type 2 diabetes mellitus or non‐alcoholic fatty liver disease (NAFLD), amongst others. In particular, experimental models of diet‐induced NAFLD often require over 16 weeks for the comprehensive manifestation of disease‐related complications, with some studies extending its duration beyond 30 weeks [20]. Similarly, while no changes were observed at 12 weeks, a significant decline in the number of myenteric neurons was detected when the duration of high fat feeding was extended to 18 and 24 weeks. Prolonging HFD exposure to 24 weeks did not result in a statistically significant further reduction in neuronal density compared with 18 weeks, although neuronal density was reduced by 17% and 22% after 18 and 24 weeks of HFD exposure, respectively. Our data are in line with previous studies reporting a similar reduction in myenteric neurons [21, 22]. Hence, future investigation should focus on unraveling how HFD‐induced low grade inflammation changes the phenotype, cell state and function of enteric neurons and their neighboring cells causing the altered gastrointestinal transit (i.e., constipation). Indeed, others have linked age‐related neurodegeneration and altered transit to a phenotypic shift of muscularis macrophages, characterized by an increase in the expression of pro‐inflammatory markers and downregulation of the transcription factor FoxO3 [12]. Also, reduced clearance of dead cells and impaired macrophage‐mediated resolution of intestinal damage was observed in HFD fed mice after DSS‐induced colitis, suggesting that elevated exposure to dietary lipids may interfere with the tissue‐supportive functions of intestinal macrophages [23]. Thus, further research is warranted in order to elucidate transcriptomic changes in gut macrophages as well as in other cell populations which may be underlying HFD‐induced enteric neuron loss.

In the present study, our findings showcase the critical importance of considering dietary composition and employing a matched control diet in studies of diet‐induced obesity, while emphasizing the urgent need of standardizing animal models in order to facilitate comparisons across research studies. Also, these findings suggest that a prolonged exposure to a HFD is required for the development of obesity‐associated alterations in the enteric network and gut transit. Future research should focus on evaluating the effect of longer periods of HFD exposure on gastrointestinal function, in addition to unraveling the mechanisms underlying the reduced enteric neuron density and colonic dysmotility during chronic obesity in NCD, LFD, and HFD‐fed animals to fully distinguish the effect of dietary fat from those of diet composition.

## Author Contributions

G.B. and N.S. led the project and revised the manuscript. G.B. conceived the study. G.B., N.S., and M.F.V. supervised the study. M.C.‐P. and N.S. designed and conducted the experiments and wrote the manuscript. I.A., Z.W., N.F., and M.D. provided excellent technical support and performed the mice genotyping throughout the experiments.

## Funding

G.B. is funded by ERC Advanced grant no. 833816‐NEUMACS. Z. Wang is supported by a PhD scholarship provided by the China Scholarship Council. M.F. Viola is supported by FWO PhD fellowship no. 11C2219N.

## Conflicts of Interest

The authors declare no conflicts of interest.

## Supporting information


**Figure S1:** Body weight and blood glucose over the course of the experiment. (A) Body weight at baseline (Week 0) and after administration of either low fat diet (LFD) or high fat diet (HFD) (Week 1–24). (B) Blood glucose at baseline (Week 0) and after administration of either LFD or HFD (Week 12–18‐24). (A, B) Data are shown as mean ± SD and *n* = 9–10 per group and were analyzed with 2 way repeated measures ANOVA with Šídák's multiple comparisons test correction.
**Table S1:** Antibodies used for immunofluorescence.

## Data Availability

The data that support the findings of this study are available from the corresponding author upon reasonable request.

## References

[1] M. Bluher , “Obesity: Global Epidemiology and Pathogenesis,” Nature Reviews. Endocrinology 15, no. 5 (2019): 288–298. 10.1038/s41574-019-0176-8 30814686

[2] P. Pecora , C. Suraci , M. Antonelli , S. de Maria , and W. Marrocco , “Constipation and Obesity: A Statistical Analysis,” Bollettino della Società Italiana di Biologia Sperimentale 57, no. 23 (1981): 2384–2388. 7337751

[3] O. H. vd Baan‐Slootweg , O. Liem , N. Bekkali , et al., “Constipation and Colonic Transit Times in Children With Morbid Obesity,” Journal of Pediatric Gastroenterology and Nutrition 52, no. 4 (2011): 442–445. 10.1097/MPG.0b013e3181ef8e3c 21240026

[4] L. Marseglia , S. Manti , G. D'Angelo , et al., “Oxidative Stress in Obesity: A Critical Component in Human Diseases,” International Journal of Molecular Sciences 16, no. 1 (2014): 378–400. 10.3390/ijms16010378 PMC4307252 25548896

[5] F. Reichardt , B. Chassaing , B. G. Nezami , et al., “Western Diet Induces Colonic Nitrergic Myenteric Neuropathy and Dysmotility in Mice via Saturated Fatty Acid‐ and Lipopolysaccharide‐Induced TLR4 Signalling,” Journal of Physiology 595, no. 5 (2017): 1831–1846. 10.1113/JP273269 PMC5330876 28000223

[6] B. G. Nezami , S. M. Mwangi , J. E. Lee , et al., “MicroRNA 375 Mediates Palmitate‐Induced Enteric Neuronal Damage and High‐Fat Diet‐Induced Delayed Intestinal Transit in Mice,” Gastroenterology 146, no. 2 (2014): 473–483. 10.1053/j.gastro.2013.10.053 PMC3920196 24507550

[7] M. Anitha , F. Reichardt , S. Tabatabavakili , et al., “Intestinal Dysbiosis Contributes to the Delayed Gastrointestinal Transit in High‐Fat Diet Fed Mice,” Cellular and Molecular Gastroenterology and Hepatology 2, no. 3 (2016): 328–339. 10.1016/j.jcmgh.2015.12.008 PMC4945127 27446985

[8] U. Voss , E. Sand , B. Olde , and E. Ekblad , “Enteric Neuropathy Can Be Induced by High Fat Diet In Vivo and Palmitic Acid Exposure In Vitro,” PLoS One 8, no. 12 (2013): e81413. 10.1371/journal.pone.0081413 PMC3849255 24312551

[9] A. Viader , E. C. Wright‐Jin , B. P. S. Vohra , R. O. Heuckeroth , and J. Milbrandt , “Differential Regional and Subtype‐Specific Vulnerability of Enteric Neurons to Mitochondrial Dysfunction,” PLoS One 6, no. 11 (2011): e27727. 10.1371/journal.pone.0027727 PMC3218017 22110743

[10] L. Ye , G. Li , A. Goebel , et al., “Caspase‐11‐Mediated Enteric Neuronal Pyroptosis Underlies Western Diet‐Induced Colonic Dysmotility,” Journal of Clinical Investigation 130, no. 7 (2020): 3621–3636. 10.1172/JCI130176 PMC7324173 32484462

[11] N. Bodi , Z. Szalai , and M. Bagyanszki , “Nitrergic Enteric Neurons in Health and Disease‐Focus on Animal Models,” International Journal of Molecular Sciences 20, no. 8 (2019): 2003. 10.3390/ijms20082003 PMC6515552 31022832

[12] L. Becker , L. Nguyen , J. Gill , S. Kulkarni , P. J. Pasricha , and A. Habtezion , “Age‐Dependent Shift in Macrophage Polarisation Causes Inflammation‐Mediated Degeneration of Enteric Nervous System,” Gut 67, no. 5 (2018): 827–836. 10.1136/gutjnl-2016-312940 PMC5565713 28228489

[13] M. Chavero‐Pieres , M. F. Viola , I. Appeltans , et al., “Magnetic Resonance Imaging as a Non‐Invasive Tool to Assess Gastric Emptying in Mice,” Neurogastroenterology and Motility 35, no. 2 (2023): e14490. 10.1111/nmo.14490 PMC10078537 36371706

[14] M. J. Dalby , A. W. Ross , A. W. Walker , and P. J. Morgan , “Dietary Uncoupling of Gut Microbiota and Energy Harvesting From Obesity and Glucose Tolerance in Mice,” Cell Reports 21, no. 6 (2017): 1521–1533. 10.1016/j.celrep.2017.10.056 PMC5695904 29117558

[15] M. von Susskind‐Schwendi , A. Dötsch , V. Haberland , et al., “Addition of Soluble Fiber to Standard Purified Diets Is Important for Gut Morphology in Mice,” Scientific Reports 13, no. 1 (2023): 19340. 10.1038/s41598-023-46331-5 PMC10630450 37935741

[16] M. A. Pellizzon and M. R. Ricci , “The Common Use of Improper Control Diets in Diet‐Induced Metabolic Disease Research Confounds Data Interpretation: The Fiber Factor,” Nutrition & Metabolism (London) 15 (2018): 3. 10.1186/s12986-018-0243-5 PMC5769545 29371873

[17] L. Antonioli , V. D'Antongiovanni , C. Pellegrini , et al., “Colonic Dysmotility Associated With High‐Fat Diet‐Induced Obesity: Role of Enteric Glia,” FASEB Journal 34, no. 4 (2020): 5512–5524. 10.1096/fj.201901844R 32086846

[18] R. C. Bortolin , A. R. Vargas , J. Gasparotto , et al., “A New Animal Diet Based on Human Western Diet Is a Robust Diet‐Induced Obesity Model: Comparison to High‐Fat and Cafeteria Diets in Term of Metabolic and Gut Microbiota Disruption,” International Journal of Obesity 42, no. 3 (2018): 525–534. 10.1038/ijo.2017.225 28895587

[19] F. Reichardt , C. Baudry , L. Gruber , et al., “Properties of Myenteric Neurones and Mucosal Functions in the Distal Colon of Diet‐Induced Obese Mice,” Journal of Physiology 591, no. 20 (2013): 5125–5139. 10.1113/jphysiol.2013.262733 PMC3810814 23940384

[20] J. M. Eng and J. L. Estall , “Diet‐Induced Models of Non‐Alcoholic Fatty Liver Disease: Food for Thought on Sugar, Fat, and Cholesterol,” Cells 10, no. 7 (2021): 1805. 10.3390/cells10071805 PMC8303413 34359974

[21] E. J. Beraldi , S. C. Borges , F. L. A. de Almeida , A. dos Santos , M. J. A. Saad , and N. C. Buttow , “Colonic Neuronal Loss and Delayed Motility Induced by High‐Fat Diet Occur Independently of Changes in the Major Groups of Microbiota in Swiss Mice,” Neurogastroenterology and Motility 32, no. 2 (2020): e13745. 10.1111/nmo.13745 31721393

[22] L. R. Rivera , C. Leung , R. V. Pustovit , et al., “Damage to Enteric Neurons Occurs in Mice That Develop Fatty Liver Disease but Not Diabetes in Response to a High‐Fat Diet,” Neurogastroenterology and Motility 26, no. 8 (2014): 1188–1199. 10.1111/nmo.12385 24952996

[23] A. A. Hill , M. Kim , D. F. Zegarra‐Ruiz , et al., “Acute High‐Fat Diet Impairs Macrophage‐Supported Intestinal Damage Resolution,” JCI Insight 8, no. 3 (2023): e164489. 10.1172/jci.insight.164489 PMC9977439 36538527

