# Supplementary material for: High Fat Diet Alters Gastrointestinal Function and Results in Reduced Enteric Neuron Density
Source: Neurogastroenterol Motil. 2026 Oct 18;38(10):e70470. doi: 10.1111/nmo.70470 (PMC13678409; doi:10.1111/nmo.70470)
Supplement: Supplementary file 1 [file NMO-38-e70470-s001.docx]

## Supplementary information

Figure S1: **Body weight and blood glucose over the course of the experiment**. **(A)** Body weight at baseline (week 0) and after administration of either low fat diet (LFD) or high fat diet (HFD) (week 1-24). **(B)** Blood glucose at baseline (week 0) and after administration of either LFD or HFD (week 12-18-24). (A-B) Data are shown as mean ± SD and *n* = 9-10 per group and were analysed with 2 way repeated measures ANOVA with Šídák's multiple comparisons test correction.

Supplementary table 1: Antibodies used for immunofluorescence

| **Antibody** | **Source** | **Cat. Number** |
| --- | --- | --- |
| Anti HuC/D | Supplied by V.Lennon (Mayo Clinic) |  |
| Donkey anti-Human Cy3 | Jackson ImmunoResearch | 709-165-149 |
